# Altered spontaneous brain activity patterns in patients with neovascular glaucoma using amplitude of low‐frequency fluctuations: A functional magnetic resonance imaging study

**DOI:** 10.1002/brb3.2018

**Published:** 2021-01-01

**Authors:** Zhi‐You Peng, Yu‐Xin Liu, Biao Li, Qian‐Min Ge, Rong‐Bin Liang, Qiu‐Yu Li, Wen‐Qing Shi, Ya‐Jie Yu, Yi Shao

**Affiliations:** ^1^ Department of Ophthalmology The First Affiliated Hospital of Nanchang University Jiangxi Province Clinical Ophthalmology Institute Nanchang China

**Keywords:** amplitude of Low‐frequency fluctuations (ALFFs), brain activity patterns, correlation, Neovascular glaucoma (NVG), rs‐fMRI

## Abstract

**Background:**

Neovascular glaucoma (NVG) can cause irreversible visual impairment and abnormal spontaneous changes in brain's visual system and other systems. There is little research on this aspect at present. However, amplitude of low‐frequency fluctuations (ALFFs) can be used as an rs‐fMRI analysis technique for testing changes in spontaneous brain activity patterns.

**Purpose:**

The aim of this study was to probe the local characteristics of spontaneous brain activity in NVG patients and analyze their correlation with clinical behaviors.

**Methods:**

Resting‐state functional magnetic resonance imaging (rs‐fMRI) scans were obtained from eighteen patients with NVG (8 males, 10 females) and eighteen healthy controls (HCs; 8 males and 10 females) who were matched in age, gender, and education level. We evaluated spontaneous brain activity with the ALFF method. A receiver operating characteristic (ROC) curve was used to compare the average ALFF values for altered brain regions of NVG patients with those of HCs.

**Results:**

Compared with HCs, NVG patients had lower ALFF values in the right cuneus, right middle occipital gyrus, left cingulate gyrus, right precuneus, and left medial frontal gyrus (*p < *0.001). Higher ALFF values were observed in the right superior frontal gyrus and left middle frontal gyrus (*p < *0.001). Analysis of the ROC curves of the brain regions showed that the specificity and accuracy of ALFF values between NVG and HCs in the area under the curve were acceptable (*p* < 0.001).

**Conclusion:**

The patients with NVG exhibited anomalous spontaneous activity in different brain regions; these finding should establish the foundation for a more comprehensive understanding of the pathological mechanisms of NVG. Furthermore, these abnormal variations in specific brain regions can be considered possible clinical indices of NVG.

## Graphical abstract

The patients with NVG exhibited anomalous spontaneous activity in different brain regions; these abnormal variations in specific brain regions can be considered possible clinical indices of NVG.

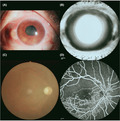

## 1 INTRODUCTION

Glaucoma is an ocular disease that causes irreversible damage to vision, and even blindness, in patients worldwide (Das, 2018). Neovascular glaucoma (NVG) is caused by severe ischemia, hypoxia, and inflammation of retinal tissue due to neovascularization on the iris surface and chamber angle (Wei et al., 2018). According to a survey, the global average NVG incidence is 6.6 per 10,000, and it accounts for 0.7%–5.1% of glaucoma cases in the Asian population (Kwon & Sung, 2017). Common causes of NVG include central retinal vein occlusion and diabetic retinopathy (Sun et al., 2016). The clinical manifestations of neovascular glaucoma include intractable hypertension, photophobia, intolerable eye pain, hyperemia, and corneal edema, with serious implications for patients’ quality of life (Wei et al., 2018). In general, the clinical pathological process of neovascular glaucoma can be divided into three stages: early glaucoma, open‐angle glaucoma, and angle‐closure glaucoma. Owing to the poor responses to treatment, the third stage of chamber angle adhesion is called refractory glaucoma (Rodrigues et al., 2016). Therefore, detecting NVG early and providing effective treatment is important.

Resting‐state magnetic resonance imaging has been widely applied in research on the central mechanisms of NVG. It is used to monitor the spontaneous activity of cerebral nerves when the subject is not performing a task, and has provided novel insight into the pathophysiology of the disease (Dai et al., 2015). Low‐frequency fluctuation amplitudes can be used as an rs‐fMRI analysis technique for measuring the regional spontaneous neuronal activity in blood oxygenation level‐dependent (BOLD) signals (Logothetis et al., 2001). Because of its reliable characterization and simple calculation requirements, the amplitude of low‐frequency fluctuation (ALFF) method is appropriate and effective for analysis of rs‐fMRI data. It has been confirmed that ALFF performed moderately to well in test–retest reliability (Dai et al., 2015; Zuo et al., 2010). ALFF is a reliable biomarker for many nervous system diseases, including Parkinson's disease (Hu et al., 2015), epilepsy (Wang et al., 2015), and schizophrenia (Zhang et al., 2018). In addition, there are many fMRI studies that have used ALFF to analyze the changes in spontaneous brain activity patterns in some ophthalmic illnesses, such as strabismus, optic neuritis, monocular blindness, retinal detachment, and retinal vein occlusion (Huang, Cai, et al., 2015; Li et al., 2016; Min et al., 2018; Shi et al., 2019; Wu et al., 2019) (Table 1).

**TABLE 1:** Summary of previous studies on the application of the amplitude of low‐frequency fluctuation method in diseases

Disease	First author	Year	Reference
Parkinson's disease	Hu	2015	Hu et al., 2015
Epilepsy	Wang	2015	Wang et al., 2015
Optic neuritis	Huang	2015	Huang, Cai, et al., 2015
Single blind	Li	2016	Li et al., 2016
Schizophrenia	Zhang	2018	Zhang et al., 2018
Strabismus	Min	2018	Min et al., 2018
Retinal vein occlusion	Wu	2019	Wu et al., 2019
Retinal detachment	Shi	2019	Shi et al., 2019

Therefore, the application of neuroimaging may be helpful in exploring the processes of brain changes in NVG patients, which may contribute to an understanding of the potential mechanisms of NVG.

## 2 MATERIALS AND METHODS

### 2.1 Participants

**FIGURE 1:**
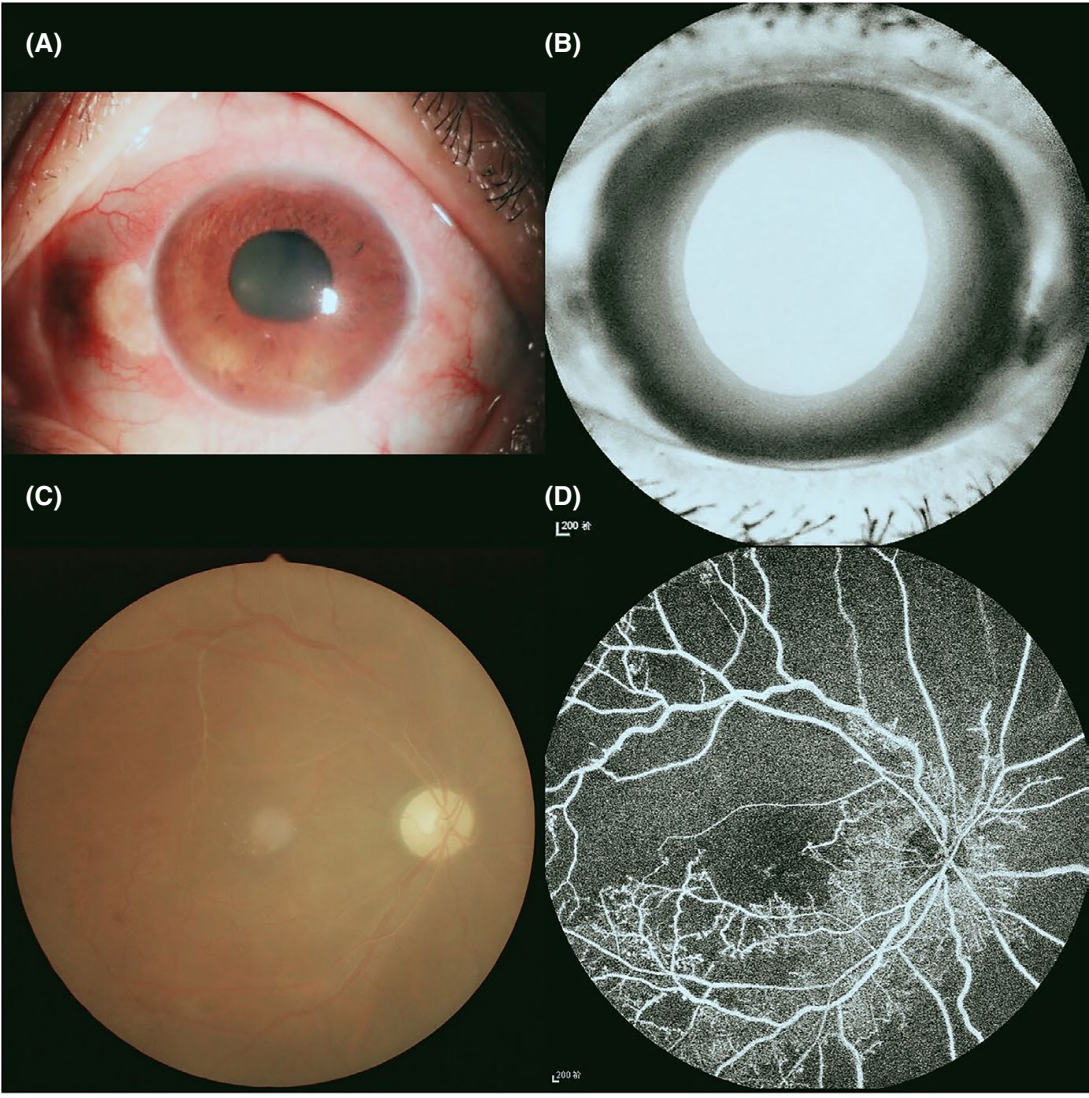
Typical example of NVG ophthalmic examination. (a) Example of NVG seen on photographic images of the anterior segment; (b) example of NVG seen on fluorescein iris angiography. Example of NVG seen on fundus images; (c) example of NVG seen on fundus‐colorized photography; (d) example of NVG seen on fundus fluorescein angiography images. Abbreviations: NVG, neovascular glaucoma

This study included 18 patients with NVG (male, *n* = 8; female, *n* = 10) from the First Affiliated Hospital of Nanchang University. The inclusion criteria were as follows: (a) the existence of new blood vessels in the iris identified in an anterior photograph (Figure 1); (b) the presence of Stage III NVG; and (c) both eyes are involved. The exclusion criteria were as follows: (a) extraocular or intraocular surgery history; and (b) diagnosis of cardiovascular diseases, mental illness, and other systemic diseases.

Eighteen healthy subjects (8 females and 10 males) who were similar in age, gender, and educational level to the patients with NVG were enrolled in the study. The exclusion criteria were as follows: (a) ocular disease history; (b) presence of malformations in the cerebral parenchyma; (c) presence of cardiovascular diseases or psychiatric diseases; and (d) presence of contraindications for MRI scanning (e.g., metal devices and pacemakers).

The study was authorized by the Medical Ethics Committee of the First Affiliated Hospital of Nanchang University. All research methods followed the guidelines of the Declaration of Helsinki. All participants were volunteers, and they were informed of the purpose, methods, and potential risks. All subjects provided informed consent.

### 2.2 MRI parameters

MRI was performed using a Trio 3‐Tesla MRI scanner (Siemens AG). The functional data were procured by applying a three‐dimensional metamorphic gradient recalled‐echo pulse sequence. The whole process was completed within eight minutes. Consequently, 240 functional images were collected with the following settings: acquisition matrix, 64 × 64; field of view, 220 × 220 mm; thickness, 4.0 mm; gap, 1.2 mm; repetition time, 2,000 ms; echo time, 30 ms; flip angle, 90°, and 29 axial. During the scan, all participants were asked to keep their eyes closed while awake and breathe normally (Huang, Zhong, et al., 2015).

### 2.3 fMRI data processing

To remove any incomplete data, we applied MRIcro software (Nottingham University). The data were preprocessed with the Data Processing Assistant for resting‐state fMRI (rs‐fMRI; DPARSFA 4.0; http://rfmri.org/DPARSF), which was based on the rs‐fMRI Data Analysis Toolkit (REST; http://www.restfmri.net) and statistical parametric mapping (SPM; http://www.fil.ion.ucl.ac.uk/spm). This processing method has been described in detail previously (Shi et al., 2019).

### 2.4 ALFF analysis

We calculated ALFF values by smoothing the remaining data with a full‑width Gaussian kernel that was 6 × 6 × 6 mm^3^ at half‑maximum. To reduce the influences of frequent physiological respiratory, low‑frequency drift, and cardiac noise, fMRI images were de‐trended and bandpass‑filtered (0.01–0.08 Hz). A fast Fourier transform (FFT) algorithm was used to convert the smoothed signals of each voxel from the time to frequency domains, and the power spectrum was obtained. ALFF maps were then differentiated using the average value of each ALFF map. Please refer to reference 18 for details of the analysis (Li et al., 2014).

### 2.5 Statistical analysis

The data of NVG patients and HCs were analyzed with spss software version 20.0 (IBM Corp.) using an independent‐sample *t* test (age and weight) and chi‐squared test (sex and handedness). ALFF Values were expressed as the mean ± standard deviation. *p* < 0.05 was the statistical threshold for the voxel level for multiple comparisons according to the Gaussian random field (GRF) theory. AlphaSim was corrected at a significance level of *p* < 0.01 and a cluster size of >40 voxels. The average ALFF values in different brain regions were used to assess differences between the two groups using a receiver operating characteristic (ROC) curve and the area under the curve (AUC), which showed the diagnosis rate. Values between 0.7 and 0.9 suggested confidence in accuracy, while values above 0.9 indicated high accuracy.

## 3 RESULTS

### 3.1 Demographics and disease characteristics

There were no significant differences in gender (*p* > 0.99), age (*p* = 0.953), and handedness (*p* > 0.99) between the patients with NVG and HCs. However, compared with HCs, there were apparent differences in best‐corrected VA‐right (*p* = 0.022), best‐corrected VA‐left (*p* = 0.024), IOP‐left (*p* = 0.009), and IOP‐right (*p* = 0.012). We also measured the highest maximal IOP of both eyes (left:right). The mean duration of NVG was 4.02 ± 1.46 years (Table 2).

**TABLE 2:** Demographics and behavioral results of NVG and HCs groups

	NVG	HC	*t* value	*p* value
Male/female	8/10	8/10	*N*/A	>0.99
Age (years)	54.56 ± 10.89	53.92 ± 9.76	0.423	0.953
Handedness	20R	20 R	*N*/A	>0.99
Duration (years)	4.02 ± 1.46	N/A	N/A	N/A
Best‐corrected VA‐L	0.27 ± 0.08	1.05 ± 0.18	5.489	0.024
Best‐corrected VA‐R	0.24 ± 0.12	0.95 ± 0.11	5.973	0.022
IOP‐L	28.88 ± 11.37	14.21 ± 479	12.842	0.009
IOP‐R	27.78 ± 13.64	15.94 ± 3.32	11.529	0.012

Notes:

Independent *t* tests comparing the age of two groups (*p* < 0.05) represented statistically significant differences. Male/female and handedness were analyzed using chi‑squared test.

Abbreviations: and IOP, intraocular pressure; HCs, healthy controls; L, left; N/A, not applicable; NVG, neovascular glaucoma; R, right; VA, visual acuity.

### 3.2 ALFF differences

**FIGURE 2:**
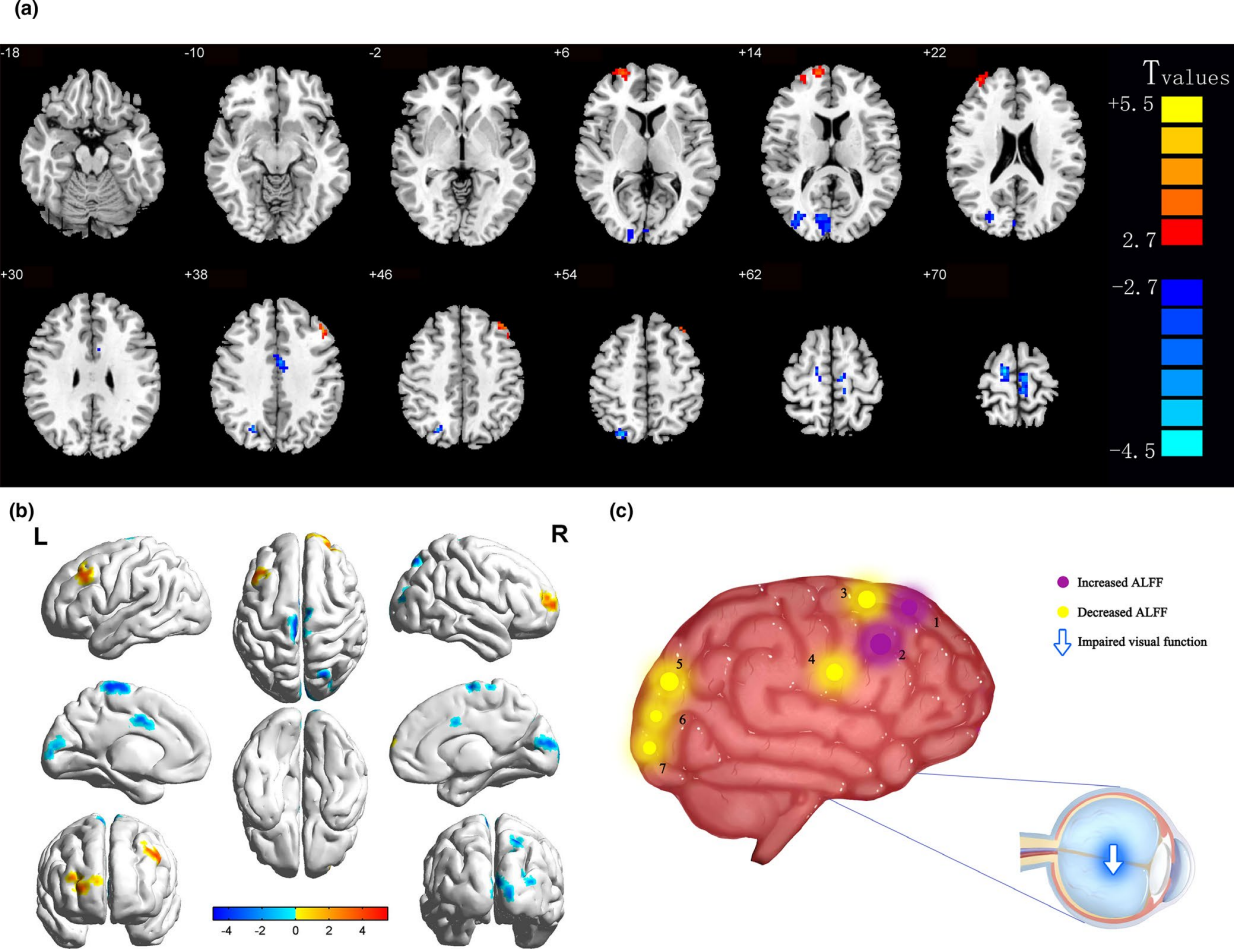
Significant differences in spontaneous brain activity between the patients with NVG and HCs. The sizes of the spots represent the degree of quantitative changes. The different brain regions were detected in the right cuneus, right middle occipital gyrus, left cingulate gyrus, right precuneus, left medial frontal gyrus, right superior frontal gyrus, and left middle frontal gyrus. The blue areas denote that patients with NVG exhibit lower ALFF values in brain areas than HCs, and the red areas denote brain regions with higher ALFF values [*p* < 0.001 for multiple comparisons using Gaussian random field theory (z. 2.3, *p* < 0.001, cluster > 13 voxels, AlphaSim corrected)]. Abbreviations: ALFF, amplitude of low‐frequency fluctuation; NVG, neovascular glaucoma; HCs, healthy controls; L, left; R, right. 1‐right superior frontal gyrus; 2‐left middle frontal gyrus; 3‐left medial frontal gyrus; 4‐left cingulate gyrus; 5‐right precuneus; 6‐right cuneus; and 7‐right middle occipital gyrus

**FIGURE 3:**
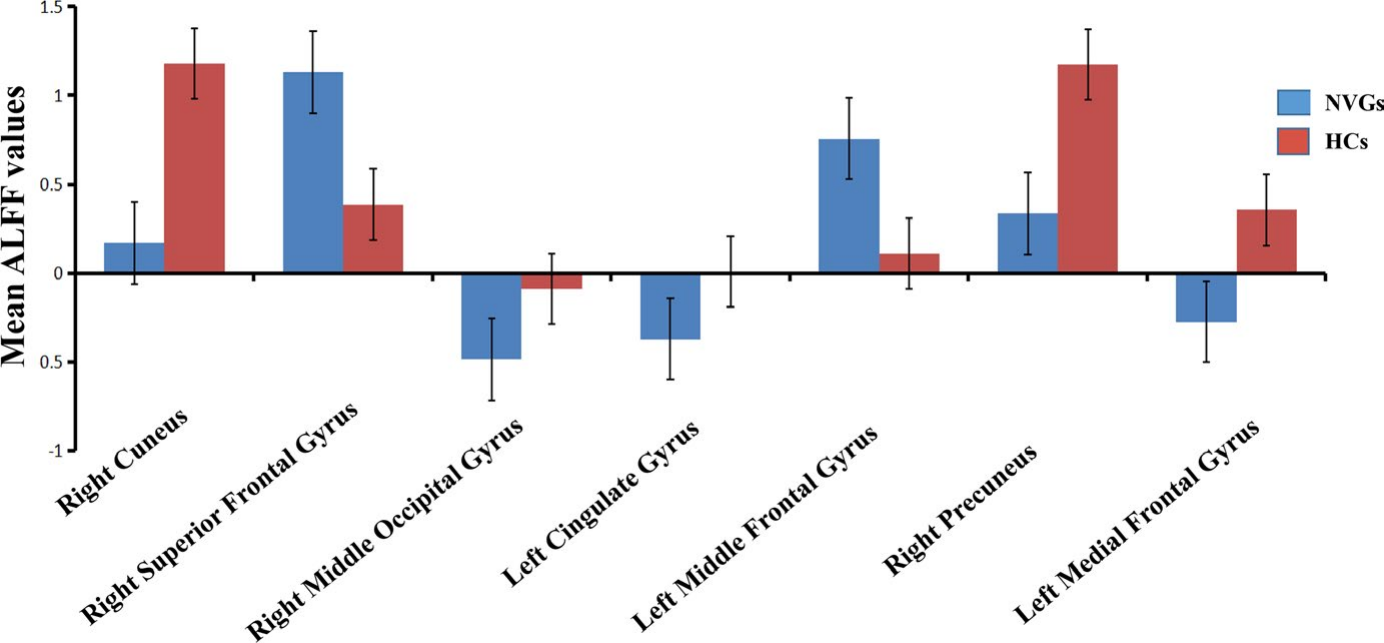
Means of altered spontaneous brain activity compared between the patients with NVG and HC groups. The statistical threshold was set at a voxel with *p* < 0.01 for multiple comparisons using family‐wise error correction (z > 2.3; *p* < 0.01; cluster, >40 voxels). ^*^ *p* < 0.05. Abbreviations: ALFF, amplitude of low‐frequency fluctuation; NVG, neovascular glaucoma; and HCs, healthy controls

Compared with the HCs, NVG patients had significantly higher ALFF values in the right superior frontal gyrus (SFG) (*p <* 0.001) and left middle frontal gyrus (MFG1) (*p < *0.001), but lower ALFF values in the right cuneus (*p < *0.001), right middle occipital gyrus (MOG) (*p *< 0.001), left cingulate gyrus (CG) (*p < *0.001), right precuneus (*p <* 0.001), and left medial frontal gyrus (MFG2) (*p <* 0.001) (Figures 2 and 3, Table 3).

**TABLE 3:** Brain areas with significantly different ALFF values between two groups

Condition	Brain regions	MNI coordinates			BA	Peak voxels	*T* value	*p*‐values
		X	Y	Z				
NVG < HCs	Cuneus .R	12	−81	15	18	94	−3.3837	*p* < 0.001
NVG < HCs	Middle occipital gyrus . R	33	−78	15	19	42	−3.5185	*p* < 0.001
NVG < HCs	Cingulate gyrus. L	−6	−9	36	24	46	−4.5442	*p* < 0.001
NVG < HCs	Precuneus. R	24	−69	42	7	44	−4.6719	*p* < 0.001
NVG < HCs	Medial frontal gyrus. L	−3	−33	75	4	154	−4.5474	*p* < 0.001
HCs < NVG	Superior frontal gyrus. R	30	63	9	10	94	4.0121	*p* < 0.001
HCs < NVG	Middle frontal gyrus . L	−45	30	42	9	53	5.5066	*p* < 0.001

Notes:

An independent‐samples *t* test was applied to get *p*‐values for comparisons between NVG group and HC group.

Abbreviations: ALFF, amplitude of low‐frequency fluctuation; and R, right; BA, Brodmann area; HCs, healthy controls; L, left; MNI, Montreal Neurological Institute; NVG, neovascular glaucoma.

### 3.3 ROC curve analysis

**FIGURE 4:**
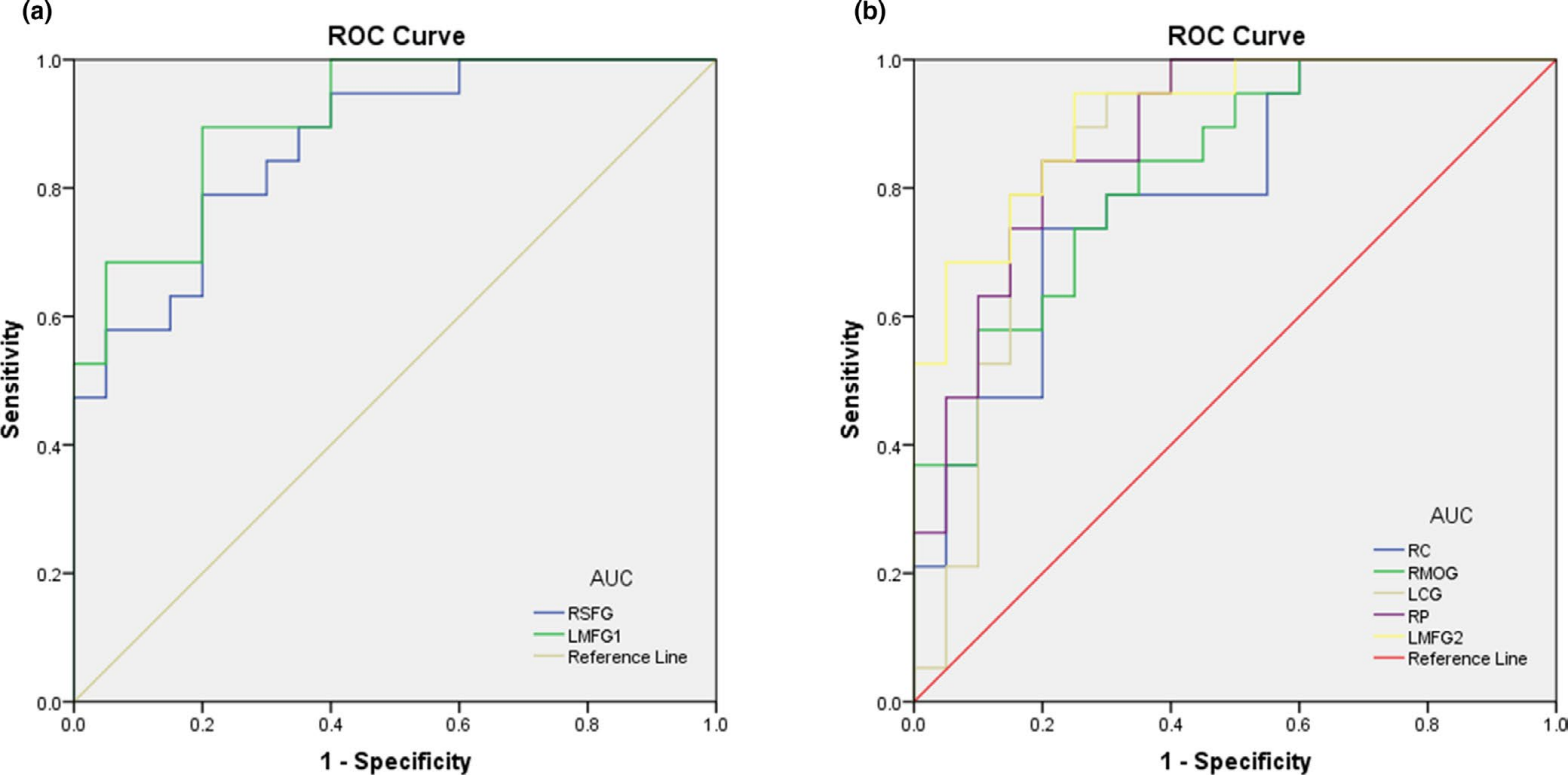
ROC curve analysis of the mean ALFF values for altered brain regions. (a) The area under the ROC curve was 0.868 (*p* < 0.001; 95% CI: 0.760–0.977) for RSFG and LMFG1 0.908 (*p* < 0.001; 95% CI: 0.820–0.996). (b) The area under the ROC curve was 0.795 (*p* = 0.002; 95% CI: 0.655–0.935) for RC; RMOG 0.826 (*p* < 0.001; 95% CI: 0.700–0.953); LCG 0.861 (*p* < 0.001; 95% CI: 0.734–0.987); RP 0.879 (*p* < 0.001; 95% CI: 0.772–0.986); and LMFG2 0.913 (*p* < 0.001; 95% CI: 0.828–0.999). Abbreviations: ROC, receiver operating characteristic; NVG, neovascular glaucoma; HCs, healthy controls; RSFG, right superior frontal gyrus; LMFG1, left middle frontal gyrus; RC, right cuneus; RMOG, right middle occipital gyrus; LCG, left cingulate gyrus; RP, right precuneus; and LMFG2, left medial frontal gyruss

The average ALFF values of the patients with NVG and HCs were analyzed using ROC curves, with a larger area under the curve (AUC) demonstrating a better diagnostic value. The AUCs for ALFF values in different brain regions were as follows: (a) NVG > HCs: right superior frontal gyrus 0.868, (*p* < 0.001; 95% CI: 0.760–0.977); left middle frontal gyrus 0.908 (*p* < 0.001; 95% CI: 0.820–0.996); (b) NVG < HCs: right cuneus 0.795 (*p* = 0.002; 95% CI: 0.655–0.935); right middle occipital gyrus 0.826 (*p* < 0.001; 95% CI: 0.700–0.953); left cingulate gyrus 0.861 (*p* < 0.001; 95% CI: 0.734–0.987); right precuneus 0.879 (*p* < 0.001; 95% CI: 0.772–0.986); and left medial frontal gyrus 0.913 (*p* < 0.001; 95% CI: 0.828–0.999; Figure 4).

## 4 DISCUSSION

In the early stages of NVG, typical microcapillary dilations are present on the surface of the chamber angle. In the stages leading to open‐angle glaucoma, these new blood vessels completely block the original iris surface structure and obstruct the outflow of aqueous humor. Finally, when angle‐closure glaucoma develops, the blood vessels contract, pulling the iris to the trabecular meshwork to form peripheral conjunctival adhesions. The fiber blood vessels on the front surface of the iris contract and the pigment layer behind the iris is pulled toward the pupil to cause pupil pigment eversion Rodrigues (GB, Abe, 2016). The cornea swells and becomes moderately to severely congested. As a result, intraocular pressure (IOP) may increase to 60 mm Hg and become refractory to reduction using drugs, such as atropin and corticosteroid eye drops. In addition, surgery tends to be ineffective and often results in serious visual impairment or even blindness. Patients also experience severe ocular pain because of increased IOP (Wei et al., 2018).

ALFF has been widely used to study primary open‐angle glaucoma (Liu et al., 2014 and Zhang et al., 2019). Here, we used ALFF to study Stage III angle‐closure NVG. We found increased neural activity in the right superior frontal gyrus and left middle frontal gyrus regions, but decreased neural activity in the right cuneus, right middle occipital gyrus, left cingulate gyrus, right precuneus, and Left medial frontal gyrus regions. Structural alterations in various regions of the brain and their potential impact are shown in discussion below and Table 4.

**TABLE 4:** Brain regions alternation and its potential impact

Brain regions	Experimental result	Main brain function	Anticipated results
Cuneus	NVG < HCs	Process visual information	Visual impairment
Middle occipital gyrus	NVG < HCs	Related to visual perception	Visual impairment
Cingulate gyrus	NVG < HCs	Part of the default‐mode network	Painful diseases and mental disorders
Precuneus	NVG < HCs	Part of the default‐mode network and related to visual function	Cognitive impairment and visual impairment
Medial frontal gyrus	NVG < HCs	Part of the default‐mode network and control spontaneous eye movements	Mental disorders
Superior frontal gyrus	HCs < NVG	Part of the default‐mode network , main premotor area, related to cognitive control and self‐consciousness	Cognitive impairment
Middle frontal gyrus	HCs < NVG	Part of the default‐mode network	Painful diseases

Note

Abbreviations: and HCs, healthy controls; NVG, neovascular glaucoma.

The frontal lobe, which is located above the lateral fissure and in front of the central sulcus, accounts for about one‐third of the brain parenchyma. The SFG, which occupies one‐third of the frontal lobe, is the main premotor area. Lesions in the anterior temporal lobe can lead to mental disorders, memory and attention loss, and delayed responses. Liu et al. (2014) found that the Hodapp–Anderson–Parrish (HAP) score of primary open‐angle glaucoma (POAG) was positively related to the ALFF values of the right SFG and that the right frontal cortex was therefore correlated with the severity of glaucoma. However, Huang, Zhong, et al. (2015) found that the ALFF values for the bilateral SFGs and MFG1 decreased in patients diagnosed with primary angle‐closure glaucoma (PAAG). In addition, Zhang et al. (2019) found that FCD values of the left MFG1 in patients with PAAG were significantly lower than those in the HC group. These results were not consistent with our results, which may be related to differences in pathogenesis and pathology between POAG and Stage III of NVG. It was shown that the SFG is related to cognitive control and self‐consciousness (Goldberg et al., 2006; Tully et al., 2014). In addition, studies have shown a notable rise in nerve activity in the MFG1 of patients with muscular and skeletal pain syndrome (Molina et al., 2017). Occlusal pain in temporomandibular joint disorders can lead to activation of the MFG1 (Brandão Filho et al., 2015). The SFG and MFG1 of patients with angle‐closure glaucoma exhibit abnormal brain activity.

A default‐mode network (DMN) is a large‐scale brain network with highly related activities. Of interest, many mental diseases, such as obsessive–compulsive disorder, are now considered to be related to aberrations in DMN (Wang et al., 2019). The highest level of overlap and consistency was found in the DMN region between the structural connections detected by diffusion MRI and functional correlations detected by rs‐MRI. This provided evidence that neurons in the DMN area are connected through large axons, which results in the correlation of activities in these areas. Many diseases that involve pain, such as dysmenorrhea and migraines, involve DMN disorders (Coppola et al., 2018; Liu et al., 2017). Patients with PAAG and multiple sclerosis have also been reported to exhibit DMN disorders (Huang, Zhong, et al., 2015; Sacco et al., 2013). The core areas of DMN are the precuneus and cingulate cortex (Sreekumar et al., 2018).

To date, there are few studies in the literature on neuroimaging and neuropsychology related to the precuneus. However, recent brain functional imaging studies have found that the precuneus is related to many high‐level cognitive functions, such as space memory, self‐related information processing, and various aspects of consciousness (Lundstrom et al., 2005; Nagahama et al., 1999). The posterior precuneus has functional connectivity with adjacent visual cortex areas (Margulies et al., 2009). The precuneus is situated in the medial wall of area BA7, which plays a key role in visual motor coordination (Le et al., 2017). The right precuneus has been associated with visual spatial cognition and visual short‐term memory (Mahayana et al., 2014; Sheremata et al., 2018). Ogiso et al. (2000) indicated that the precuneus also participates in motion image perception, and transmits motion signals to the supplementary motion area. Shi et al. (2019) showed that the ALFF values of the right precuneus were decreased in PAAG patients; therefore, decreased neural activity of the precuneus indicates impairment of visual function. In addition, individual differences in pain sensitivity have been confirmed to be related to the precuneus (Goffaux et al., 2014).

The cuneus has the ability to process visual information (Vanni et al., 2001). Shao et al. (2018) found that the cuneus had an abnormal functional connection in patients with monocular blindness (MB). Li et al. (2016) found that the ALFF values of the cuneus were decreased in patients with MB. In this study, the significant decrease of ALFF values in the cuneus of NVG suggests that the visual impairment caused by NVG is closely related to the abnormal function of the cuneus. In addition, previous study demonstrated that the right cuneus had abnormal brain activity in patients with acute anxiety disorder (Lai et al., 2013). Therefore, we speculated that the abnormal activity of cuneus may also be related to the emotional disorder caused by the pain and visual impairment caused by NVG.

Zhang et al. (2018) found that changes in brain activity occur in the MOG in a resting state in patients with mental disorders. Hummel et al. (2013) confirmed that the MOG on the right side is related to visual perception. Zhang et al. (2015) showed that the gray matter volume of the left medial frontal gyrus in POAG patients is significantly reduced. A previous study showed that the cingulate gyrus is closely related to emotion, pain cognition, spatial information processing, and long‐term memory (Kobayashi et al., 2011). The results of this experiment showed that the ALFF values of these brain regions in NVG patients were significantly reduced, which indicated the existence of dysfunction. Besides, the patients often show visual abnormalities, pain, and emotional disorders. The experimental data and clinical manifestations are consistent with above conclusions.

One limitation of the ALFF method is that it focuses on low‐frequency (usually < 0.1 Hz) BOLD signals, and sensitivity decreases when brain nerve activity is complex (Wurina et al., 2012). The principle of fMRI is dependent on blood oxygen levels, which reflect brain function activity, and involves measurement of changes in magnetic resonance signals. These signal changes are caused by the changes in brain blood flow and cerebral blood oxygen levels as a result of nerve activity, which is one of the classical algorithms for resting data analysis. ALFF, to some extent, indicates the strength of neuronal activity; however, this method is limited in that signals are easily affected by external and physiological noise, which affects data analysis of the ventricular system. However, in fractional ALFF (fALFF), the non‐specific signals of the ventricles are effectively inhibited and interference due to physiological noise is reduced. Therefore, fALFF is a standardized ALFF at the individual level that enables the disadvantages of ALFF to be effectively circumvented, and improves the specificity and sensitivity of detection (Qiu et al., 2019).

There are several limitations to this study. First, the number of available subjects was limited, which may lead to low reproducibility of results and overestimates of effect size (Button et al., 2013). Second, severe clinical manifestations of NVG may lead to abnormal brain changes due to psychological and mental factors (Sato et al., 2019). In addition, there are inevitable individual differences. Finally, because the patients recruited in this study were all at Stage III, we did not examine the abnormal spontaneous brain activity caused by NVG at Stages I and II. We will aim to investigate these aspects in future research.

## 5 CONCLUSION

Analysis of rs‐MRI data by the ALFF method revealed abnormal neural activity in specific brain areas of NVG patients. The findings may provide new insights for the study of the pathological mechanisms of NVG and new useful indicators for clinical diagnosis.

## CONFLICT OF INTEREST

This was not an industry‐supported study. The authors report no conflicts of interest in this work.

## AUTHORS' CONTRIBUTIONS

PZY and SY designed the current study. LYX and LB recruited healthy controls. GQM and YYJ performed MRI scanning. LRB, LQY, and WQS collected and analyzed the data. PZY wrote the manuscript. All the authors read and approved the final manuscript.

### Peer Review

The peer review history for this article is available at https://publons.com/publon/10.1002/brb3.2018.

## Data Availability

The datasets used and/or analyzed during the present study are available from the corresponding author on reasonable request.
